# Comparison of two surgical techniques (HOO vs. BSSO) for mandibular osteotomies in orthognathic surgery—a 10-year retrospective study

**DOI:** 10.1007/s10006-022-01073-y

**Published:** 2022-05-20

**Authors:** Lukas B. Seifert, Christopher Langhans, Yakub Berdan, Sophie Zorn, Michelle Klos, Constantin Landes, Robert Sader

**Affiliations:** 1 Department of Oral, Cranio-Maxillofacial and Facial Plastic Surgery, University Hospital Frankfurt, Goethe University, Theodor-Stern-Kai 7, 60596 Frankfurt, Germany; 2 Department of Oral, Maxillofacial and Facial Plastic Surgery, Sana Klinikum, Starkenburgring 66, Offenbach am Main, Germany

**Keywords:** Orthognathic surgery, Jaw surgery, High oblique sagittal osteotomy, Bilateral sagittal split osteotomy

## Abstract

**Purpose:**

To retrospectively compare the high-angled sagittal split osteotomy (HOO) and the bilateral sagittal split osteotomy (BSSO) for the correction of skeletal dysgnathias regarding intra- and postoperative complications.

**Methods:**

The electronic medical records of all patients treated with an orthognathic surgery at the Department for Oral, Maxillofacial and Facial Plastic Surgery, University Hospital Frankfurt, Germany, between the years 2009 and 2019 were retrospectively reviewed.

**Results:**

Two hundred ninety-one patients were included. The overall complication rates were 19.78% (BSSO) compared to 12.5% (HOO) (*p* = 0.14). Significant differences were found regarding the operation time (HOO < BSSO, *p* = 0.02), material failure (HOO > BSSO, *p* = 0.04), and early recurrence requiring revision surgery (HOO < BSSO, *p* = 0.002). The use of a ramus plate significantly reduced the risk of plate failure (2.8% < 13.6%, *p* = 0.05). More bad splits (*p* = 0.08) and early sensory disorders (*p* = 0.07) occurred in the BSSO group.

**Conclusion:**

The HOO presents a possible alternative to the BSSO since newly developed osteosynthesis material significantly reduces the risk of material failure. The BSSO is accompanied by higher risks of developing complications like a bad split and sensory disorders but, however, remains the standard for large anterior–posterior transpositions of the mandible.

## Introduction

**Fig. 1:**
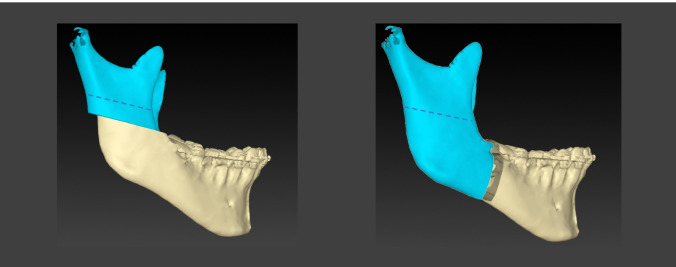
Osteotomy designs of the high-angled oblique osteotomy (left) and bilateral sagittal split osteotomy (right). The detached lines show the osteotomy on the lingual side

Today, the most frequently used surgical technique to correct a skeletal dysgnathia of the lower jaw is the bilateral sagittal split osteotomy (BSSO) (Fig. 1), which was first described by Obewegeser and then modified by Dal Point in 1961. [1] Due to the extensive bone apposition surface of the osteotomised fragments, this surgical technique especially enables good bone healing and, thus, stable surgical results. [2] The greatest disadvantage of this technique, however, is the anatomically related risk of damage to the inferior alveolar nerve in the course of the sagittal split in BSSO, which has been widely studied in the literature [3, 4, 5] and is reported to range from 11.7 to 24% of cases. [6]

An alternative to the BSSO is the high-angled sagittal split osteotomy (HOO) (Fig. 1) that was first described by Schlossmann in 1922 and later published by Perthes (1924). [7] The small bone apposition area in the HOO and the resulting delayed bone healing and lower stability as well as concerns about the proper positioning of the proximal segment were reasons why this method did not prevail for a long time. Today, mainly due to technical innovations and advances in the field of osteosynthesis [8] and osteotomy technologies, the HOO is increasingly applied again. [9] Recently, many authors have described modifications to the technique, i.e. an intraoral approach beginning at the lateral cortex, at the level of the alveolar foramen in a 45° angle with respect to the mandibular ascending branch [2] or the use of piezo-surgery resulting in better bone healing because of less intervened bone surfaces. [10,11]

There is still a lack of long-term studies with regard to intra- and postoperative complications that adequately compare the two techniques. Since orthognathic surgery is a highly selective intervention that has a great influence on the physical and psychological quality of life of the patient, undesirable results represent a serious problem. The aim of this study, therefore, was to examine and compare the two osteotomy techniques, BSSO and HOO, for correction of the lower jaw, especially with regard to the occurrence of intra- and postoperative complications.

## Materials and methods

The electronic medical records of all patients who underwent orthognathic surgery between 2009 and 2019 at the Department for Oral, Maxillofacial and Facial Plastic Surgery, University Hospital Frankfurt, Germany, were retrospectively evaluated in anonymised form. The patient collective included was composed of patients in whom a mandibular corrective osteotomy according to Obwegeser/Dal Pont (BSSO) or by a high-angled sagittal osteotomy (HOO) was performed.

Factors that led to exclusion of the study were surgical distraction therapy or temporomandibular joint replacement prior to orthognathic surgery, the presence of a systemic disease (muscular dystrophy, myasthenia gravis, osteogenesis imperfecta), a pathologic craniofacial syndrome (Crouzon syndrome, Apert syndrome, Goldenhar syndrome, etc.), symptoms of a cranio-mandibular-dysfunction (CMD) prior to orthognathic surgery and patients that underwent “surgery first” therapy without adequate orthodontic pretreatment.

The target variables recorded were gender, age, year of operation, diagnosis, duration of operation, surgical technique, used osteosynthesis material, time until the material was removed and the occurrence of intra- and postoperative complications. In terms of osteosynthesis material, a distinction was made between conventional mini-plates (X, Y, straight), individual ramus plates, screw osteosynthesis, and combined plate and screw osteosynthesis. In the case of intraoperative complications, particular attention was paid to the occurrence of bad splits as irregular fractures in the lower jaw, visible nerve damage in the course of the operation, and intraoperative bleeding. Bleedings were only recorded when they were particularly difficult to control or resulted in further surgical intervention due to a resulting hematoma.

In the case of postoperative complications, temporomandibular joint complaints, neurosensory disorders, osteosynthesis material failure, secondary bleeding, infections, and early recurrences were recorded as additional parameters. Only those cases were recorded as early recurrences that could not be corrected in the course of orthodontic therapy and that required revision surgery. In the case of osteosynthesis material, a distinction was made between a fracture of the osteosynthesis material and other stability problems of the osteosynthesis material, such as bending, loosening, a resulting pseudarthrosis, or non-union of the bone segments. In the case of postoperative neurosensory disorders, only those that were not reversible within 12 months were considered as permanent. These were included as complications in the overall complication rate. In the case of temporomandibular joint complaints, it was checked whether postoperative temporomandibular joint complaints or CMD symptoms such as postoperative pain symptoms or a restricted mouth opening of less than 2.5 cm lasting more than six (6) weeks postoperatively occurred. In the case of postoperative infections and bleeding, only those cases that required a surgical procedure were taken into account.

### Statistical evaluation

The statistical evaluation of the data collected in Excel was carried out using the statistical programs SPSS (version 27, International Business Machines Corporation, Armonk/USA) and BiAS (version 11.12, Ackermann, Frankfurt/Germany). Graphic representations were created in SPSS and Excel. For the descriptive analysis of the data set, descriptive data such as the arithmetic mean, minimum value, maximum value, the median, and the standard deviation (SD) were calculated. The values were checked for normal distribution using the Shapiro–Wilk test. If a normal distribution could not be assumed, the Mann–Whitney *U* test was used; otherwise, the parametric *t*-test was used. The level of significance was set at *p* < 0.05. To compare two categorical variables, Pearson’s chi-square test was carried out, with a Yates correction being made for case numbers below 50. If the expected frequencies were too low (< 5), Fisher’s exact test was used.

## Results

### Patient collective

A total of 219 patient cases (*n* = 219) who met the inclusion criteria were examined. In 91 patients, orthognathic surgery was performed using the BSSO and in 128 patients the HOO was used. Of the 219 patients, 126 (57.5%) were female and 93 (42.4%) were male.

The average age of all recorded patients at the time of the operation was 25.2 years, with the youngest patient being operated at 15 years and the oldest patient at 64 years (SD = 9.17). The median was 22 years. The gender-dependent age distribution showed a mean age of the female patients of 25.8 years (SD = 9.13 and median: 23 years) and for male patients 24.4 years (SD = 9.21 and median 21 years).

In the BSSO group, the mean age at the time of surgery was 25.4 years, with the youngest patient being operated at the age of 15 years and the oldest patient at the age of 64 (SD = 9.91 years, median 22 years). In the HOO group, the mean age of the patients at the time of surgery was 25.1 years. Here, the youngest patient was operated at 15 years of age and the oldest patient at 57 years of age (SD = 8.64 years, median 22 years). Over 75% of the patients were under 30 years of age at the time of the operation.

In the present patient collective, there was a skeletal class III in 126 (57.5%) cases, while a skeletal class II was found in 86 (39.2%) patients. In 30 (13.7%) cases, there was a vertical deformity, in 28 (12.7%) cases an open bite, and in 20 (0.9%) cases a deep bite. A total of 13 (5.9%) patients were affected by.

### Surgical intervention

Overall, a bimaxillary osteotomy was performed in 75.8% of cases to correct the dysgnathia. The Le-Fort I osteotomy was used as the standard procedure in the upper jaw, while the BSSO or HOO osteotomies were used in the lower jaw. In 24.2% of the patients, the dysgnathia could be surgically corrected by a monomaxillary operation of the lower jaw (BSSO or HOO). In the BSSO group, a monomaxillary osteotomy was performed significantly more often than in the HOO group (*p* = 0.023).

The mean duration of the operation was 350 ± 97.5 min for bimaxillary osteotomies and 228 ± 54.4 min if a monomaxillary osteotomy was performed.

When comparing the two surgical procedures with regard to the duration of the operation, only monomaxillary interventions were taken into account. The duration of the operation was significantly shorter for the HOO group compared to the BSSO group (*p* = 0.023). The average operating time in the HOO group was 213 ± 67.8 min (median 212 min), the minimum being 125 min. In the BSSO group, the mean duration of the operation was 240 ± 38.6 min (median 233 min) with the minimum being 185 min.

**Fig. 2:**
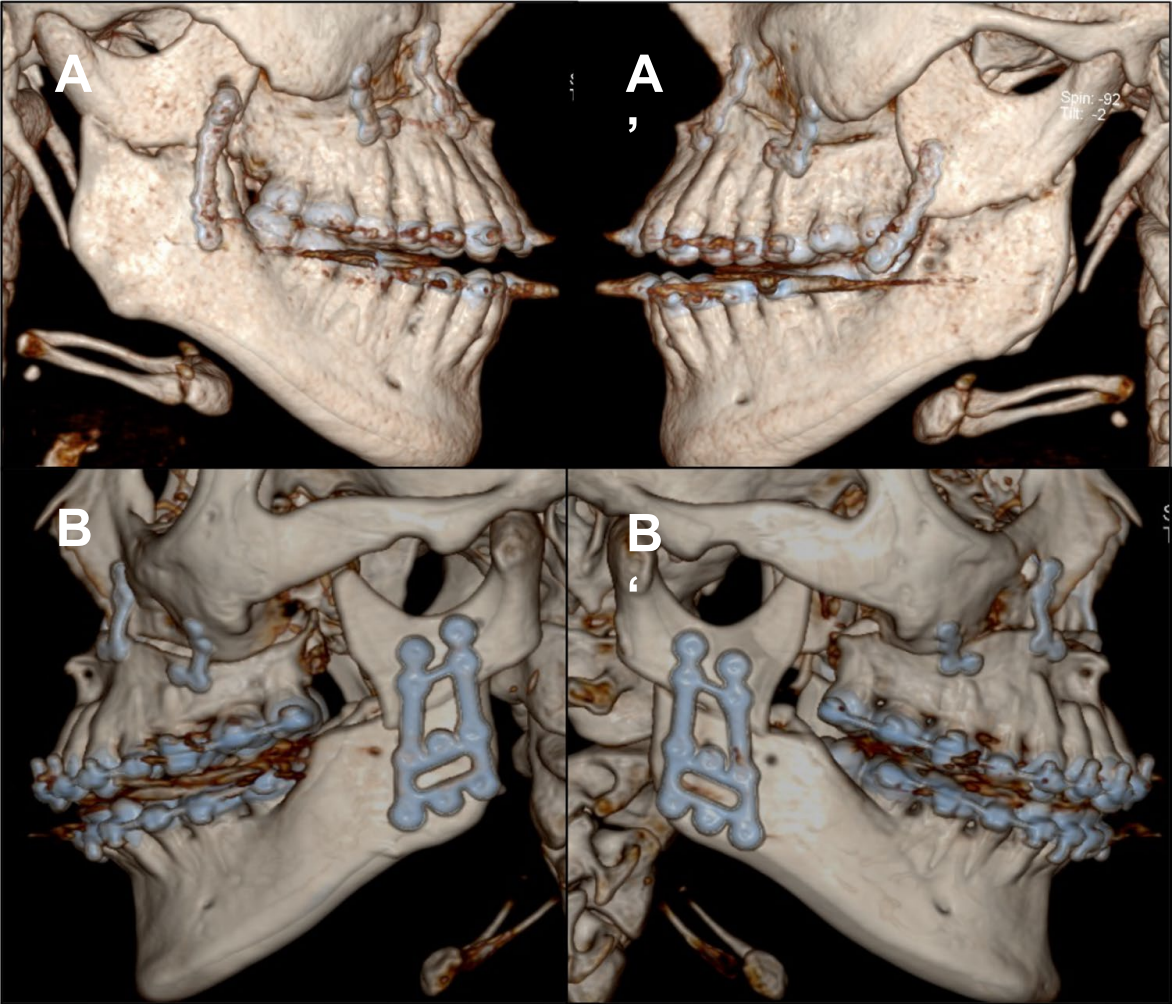
Three-dimensional presentation of a high-angled oblique osteotomy (HOO). **A**: HOO with loosened osteosynthesis material (classic straight mini-plate). **B**: HOO with dedicated ramus plate

When analysing the osteosynthesis material used, it was noticeable that both the classic mini-plate osteosynthesis and the ramus plate osteosynthesis were used in patients who were operated on with the HOO procedure (Fig. 2A and B). While classic mini-plates (X, Y, straight) were used in all patients operated with HOO in 2009 (*n* = 11), the proportion was only 50% in 2010, so that of 22 patients operated with HOO, 11 received a ramus plate. In 2011, ramus plate osteosynthesis was already used in 23 of the 24 patients operated on with HOO (Fig. 2B), and from 2012 only ramus plates were used in osteosynthesis for the HOO.

**Fig. 3:**
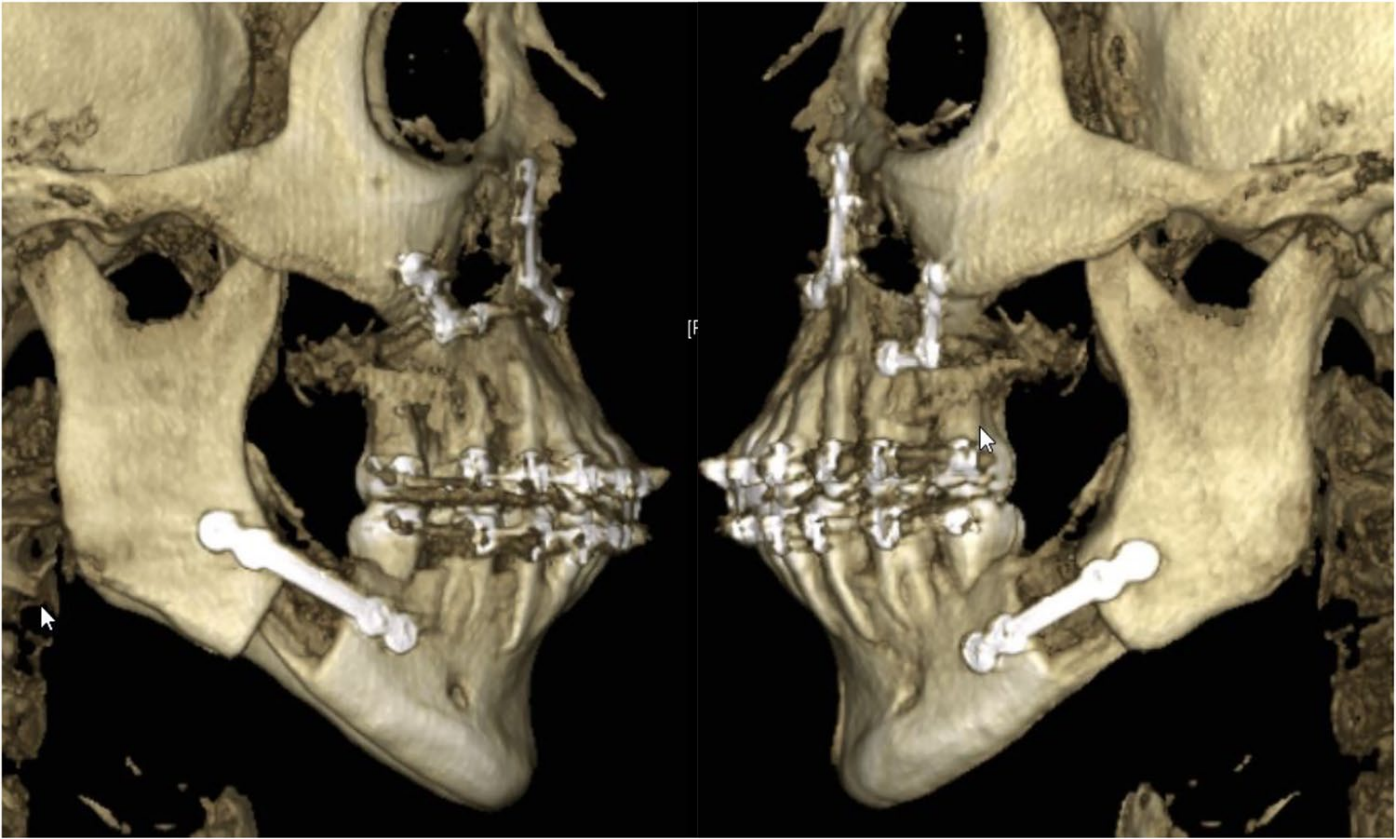
Three-dimensional presentation of a bilateral sagittal split osteotomy (BSSO) with classic straight mini-plates being used

In contrast, with the BSSO, the osteosynthesis was carried out with conventional mini-plates over the entire period observed (Fig. 3), with a combined plate and screw osteosynthesis being used in one case.

The osteosynthesis material was removed in 183 cases (79.5%) when considering the entire patient collective. In the BSSO group, 79 of the 91 patients (86.8%) and of 104 of the 128 patients (81.3%) in the HOO group had surgical material removed. The mean time to material removal in the BSSO group was 11.6 ± 4.98 months, the minimum being 3 months and the maximum being 34 months (median 12 months). In the HOO group, the period between surgery and removal of the osteosynthesis material was 10.2 ± 4.97 months, which was significantly shorter (*p* = 0.003). The minimum was 4 months and the maximum was 40 months (median 9 months). The mean follow-up time was 11.9 ± 5.2 months in the BSSO group and 10.6 ± 6.2 months in the HOO group.

### Major complications

Overall, complications occurred in 34 of the 219 patients (15.53%), while 185 patients (84.47%) had no complications.

A bad split occurred in six cases (2.7%) and clinically relevant bleeding occurred in three cases (1.4%). Postoperatively, newly developed temporomandibular joint complaints appeared in eight cases (3.7%), in seven cases an early relapse that required revision occurred (3.2%), in six cases the osteosynthesis material failed (2.7%), and in three cases there were lasting sensory disorders (1.4%). Furthermore, there was an infection postoperatively in two cases (0.9%) and in one case relevant re-bleeding (0.5%). In five (5) further cases, there were complications which, due to their rarity, were grouped under the variable “other” (2.3%). These included a postoperative lactic acidosis in a type I diabetes patient as well as a postoperative hearing loss complained of by one patient and which had existed since the operation. Likewise, the case of a compartment syndrome in the lower leg that occurred postoperatively during the inpatient stay, which required surgical intervention, was assigned to the “other” complications. A postoperative fever was documented in another patient and, in one case, a mandible fracture occurred in the previously osteotomised area after removal of the osteosynthesis material, so that these cases were also classified under “other”.

### Comparison of complications and surgical technique

In the BSSO group, the complication rate was 19.78% (18/91 patients), while it was 12.5% (16/128 patients) in the HOO group (*p* = 0.14). Table 1 gives an overview of the complication rates divided into the surgical procedures.

**Table 1:** Overview of the complications that have occurred in the BSSO and HOO groups

Complications	BSSO	HOO	*p* value
Bad split	5	1	0.084
Intraoperative bleeding	1	2	1
Plate failure	-	6	0.042
TMJ disorders	5	3	0.28
Recurrence requiring revision	7	-	0.002
Infection	1	1	1
Sensory disorders	3	-	0.07
Postoperative bleeding	1	-	0.41
Other complications	1	4	0.4
Total	24	17	

#### Bad splits

Intraoperatively, five patients (5.49%) who were operated on with the BSSO had a bad split. In contrast, a bad split occurred in only one case (0.78%) of patients who were operated on with the HOO (*p* = 0.084).

#### Intraoperative bleeding

In the BSSO group, there was a relevant intraoperative bleeding in one case (1.1%), whereas in the HOO group, two patients (1.56%) had such a complication (*p* = 1).

#### Material failure

While there was no plate failure in any patient who was operated on with the BSSO, such a complication occurred in six cases (4.69%) of the HOO group. Plate failure occurred significantly more frequently with the HOO group compared to the BSSO group (*p* = 0.042).

On closer analysis of the osteosynthesis material used, it is noticeable that, of the six plates in which the osteosynthesis material failed, three were conventional mini-plates (Fig. 2A) and three were ramus plates (Fig. 2B). In relation to the total number of osteosynthesis plates used for the HOO, the complication rate for plate failure is 13.63% for conventional mini-plates and 2.83% for ramus plates (*p* = 0.05).

In two cases, it was a plate fracture, which occurred in both cases with the conventional mini-plate and which resulted in a pseudarthrosis in one case. In the remaining four cases, the osteosynthesis plates were bent, loosened or partially lost, resulting in pseudarthroses or non-unions. This made revision surgery necessary in four of the six cases.

#### Temporomandibular joint disorders

In patients who were operated on with the BSSO, newly developed temporomandibular joint (TMJ) problems occurred in five cases (5.49%), whereas in the HOO group, this complication was only observed in three cases (2.34%) (*p* = 0.28).

This resulted in pain or a restriction of the mouth opening, whereby one case of TMJ osteoarthritis, one case of disc rupture, and one case of disc degeneration could be diagnosed by imaging. Those were treated with arthroscopy and physiotherapy.

#### Early recurrences requiring revision surgery

In seven cases (7.69%), the BSSO group had an early recurrence requiring revision surgery. However, in the majority of these cases, the incorrect positioning became visible immediately postoperatively, for example, through a frontal open bite; in one case, the indication for revision surgery was made 10 weeks postoperatively due to the active tongue pressure of a patient and an associated frontal end-to-end bite. The occurrence of this complication was significantly more frequent in the BSSO group than in the HOO group, in which this complication was not recorded (*p* = 0.0018).

#### Infections

In a total of two cases, there was a postoperative infection in the lower jaw, one of which was an intraoral fistula and an abscess in the other. In both cases, it was necessary to remove the osteosynthesis material. One infection occurred in the BSSO group (1.1%), the other in the HOO group (0.78%) (*p* = 1).

#### Sensory disorders

Postoperatively, 44 patients (48.35%) in the BSSO group were affected by sensory disorders within the first 3 months, although after 3 months only 15 patients were affected (16.48%) and after 6 months only 12 patients (13, 19%) were affected. In three patients (3.3%), the sensory disorders were not reversible 12 months after the surgery. In the HOO group, five patients (3.9%) suffered from sensory disorders within the first 3 months, four cases showed to be regredient after 3 months, and only one case (0.78%) presented a sensory disorder after 6 months. After 12 months, none of the patients in the HOO group had sensory disorder. Overall, significantly fewer sensory disorders occurred postoperatively in the HOO group compared to the BSSO group (*p* = 0.00). However, in the long term, only three cases in the BSSO group and zero cases in the HOO group were accounted as irreversible sensory disorder, which was not significant (*p* = 0.07).

#### Postoperative bleeding

In the BSSO group, there was one case (0.78%) of relevant postoperative re-bleeding, whereas there was no such complication in any patient in the HOO group (*p* = 0.41).

#### Other complications

In the HOO group, there were four cases (3.13%) of other complications, including a hearing loss, a postoperative lactic acidosis, a postoperative fever, and a mandible fracture, which occurred after removal of the osteosynthesis material. In the BSSO group, one case (1.1%) was assigned to the other complications in which postoperative compartment syndrome on the lower leg had to be treated surgically (*p* = 0.4).

## Discussion

The aim of this study was to examine and compare the two osteotomy techniques, BSSO and HOO, for correction of the lower jaw, especially with regard to the occurrence of intra- and postoperative complications. Overall, our results show that there are significant differences regarding intra- and postoperative complications rates between the BSSO and HOO.

### Patient collective

The average patient age found in this study (BSSO 25.4 years; HOO group 25.1 years) is in line with the results of comparable studies. [12,13] The optimal time of orthognathic surgery in terms of growth-related recurrences of too early interventions is still a controversial discussion in the literature [14,15]; however, a broad consensus exists that, especially in moderate deformities, maxillo-facial growth should be completed before surgery. [16]

There are, however, individual exceptions like distinct skeletal disharmonies, severe functional impairment, or a high degree of psychological strains that can justify an earlier correction. [16]

Noticeably, the majority of our patients (57.5%) were female. A possible explanation for this gender imbalance might be that, especially in female patients, a main motivation for undergoing orthognathic surgery, besides functional reasons, is an improvement of appearance and facial aesthetics. [17]

### Choice of surgical technique in comparison

Dysgnathias rarely develop in one jaw only, which often makes a correction of both jaws necessary to obtain ideal functional and aesthetic results. [18,19] However, this does not explain the significant differences regarding a mono- or bimaxillary approach (HOO 82%; BSSO 67%; *p* = 0.023) found in this study. Especially in large anterior–posterior shifts, the resulting small bone apposition surface can be a limiting factor when performing a HOO [2,8]. In a computational study, Moehlenrich et al. compared the HOO and BSSO for mandibular set-backs and advancements up to 10 mm and found that the bone apposition area reduces up to 202 mm^2^ for a mandibular set-back and to 193 mm^2^ for a mandibular advancement of 10 mm in the HOO compared to 1099 mm^2^ and 966 mm^2^ in the BSSO. Based on this, the BSSO is preferable especially in larger anteroposterior movements. [2] However, in individual cases, a larger anterior–posterior shift can be accomplished without compromising the stability of the osteosynthesis.

Also, clockwise and counter-clockwise rotations to correct vertical deformities like an open bite in a patient with an angle class II can result in limited bone apposition after osteotomy. However, this can be prevented by adapting the osteotomy design which then has to be angled from posterior-inferior to anterior–superior along the ramus. Once the mandible is advanced, the open bite will be closed without creating a large gap at the osteotomy [34]. Due to the recent advancements in virtual surgical planning including the production of individual cutting guides and osteosynthesis plates, also complex and angled osteotomy designs to achieve mandibular rotational movements are now more predictable and safer to perform. [20]

Overall, the increasing risk of impaired bone healing and recurrence might explain why a bimaxillary surgery was performed significantly more often in the HOO group.

With an average time of 213 min, the duration of surgery was significantly shorter in the HOO group. Other studies came to similar results [21,22]. Many argue that, due to the less complex osteotomy design, the HOO is quicker to perform. A shorter time of surgery is correlated with less blood loss [23] and fewer postoperative infections [24], which has to be considered especially when treating patients with previously known illnesses.

### Intraoperative complications

The reported percentage of intraoperative complications varies widely from 4.4 to 14.3% due to different study designs and surgical techniques used. [25] With eight patients (3.7%), our intraoperative complication rate seems to be at the lower end of reported rates.

#### Bad splits

The danger of a bad split, especially in the BSSO, is well-described in the literature and ranges between 0.8 und 10.9% per side [26,27], which is in line with our results (5.49%). In contrast, there is very little information on the incidence of a bad split in the HOO. In this study, only one patient (0.78%) experienced a bad split. In accordance, many authors assess the risk of a bad split in the HOO as very low due to the easy-to-perform osteotomy and its location near the ramus region of the mandible. [9,21] The individual anatomy of the ramus region is one important factor in the occurrence of a bad split. Wang et al. found that a shorter ramus and a low thickness of the buccolingual area distal to the second molar had a higher risk for a bad split. [28] Today, three-dimensional imaging has helped to preoperatively measure the width of the ramus region above the lingula and thus better assess the risk of a bad split in the HSSO and BSSO.

Even though no significant differences (*p* = 0.084) between both osteotomies were found, our results support that risk for a bad split is higher in the BSSO compared to the HOO.

#### Intraoperative bleeding

Significant intraoperative bleeding is mostly described in conjunction with the Le Fort 1 osteotomy and, thus, not connected to the BSSO or HOO. However, vessels like the retromandibular vein can lead to extensive bleeding when damaged due to osteotomies of the mandible [29], which can be avoided by using appropriate instruments (Obwegeser channel retractor) to protect the neurovascular bundle and retromandibular vein. Overall, the results of this study (BSSO 1.1%; HOO 1.5%) suggest that the risk of an intraoperative bleeding is equally high for the BSSO and the HOO.

### Postoperative complications

A total of 13.2% (HOO 10.9%; BSSO 16.5%) of the patients were affected by postoperative complications.

#### Early recurrences in need of revision

The literature distinguishes between an early recurrence which can result from an incorrect re-fixation or joint positioning [30] and a late recurrence, which usually manifest themselves in the first 2 years after surgery and can result from unexpected postoperative jaw growth; problems with neuromuscular adaptation as well as condylar resorption processes are also discussed. [30]

Interestingly, all cases of early recurrences in this study occurred in the BSSO group (*p* = 0.0018). A possible explanation for the significant differences found might be the intraoperative joint positioning, which many authors describe as the most difficult part of BSSO surgery due to lack of optical control of the condyle-fossa relationship. [31] Navigation- and computer-assisted systems as well as sonographical-assisted positioning of the condyles are discussed as possible solutions; however, the use of those devices remains uncertain and most surgeons still prefer a manual positioning. [32] In our study, all inserting ligaments and muscles were carefully detached from the coronoid process by blunt preparation to prevent an upward pulling of the proximal segment and the position of the proximal segment and the condyle was sonographically assisted in the HOO group due to the smaller proximal segment. This indicates that the intraoperative use of sonography might help to prevent an early recurrence in osteotomies of the mandible.

#### TMJ complaints

Studies report that biomechanical stress on the temporomandibular joint structures during the surgical procedure is caused by the sagittal splitting, especially in the BSSO procedure, since rotational forces exerted on the temporomandibular joint at the splitting point can lead to pressure of the condyle in its fossa. [33] Manual repositioning of the proximal segment and the subsequent osteosynthesis can also result in compression of the disc-condyle complex. [33] In the literature, an incidence between 0% and 4.3% (HOO) [6,34] and 16.3% (BSSO) [35] of newly developed TMJ complaints can be found. With 5.5% in the BSSO group and 2.3% in the HOO, comparatively few patients developed postoperatively temporomandibular joint (TMJ) complaints in the form of pain or a restriction of the mouth opening. However, this difference can be explained by the exclusion of patients that already presented TMJ complaints prior to surgery within our study design.

#### Osteosynthesis material failure

For the BSSO, the risk of material failure is reported to be very low due to the bigger bone apposition area. [36] In a computational study, Möhlenrich et al. found that a significantly larger bone apposition area can be achieved for the BSSO compared to the HOO and, thus, recommend the BSSO, at least for longer displacement distances. [2]

Interestingly, only the conventional mini-plate osteosynthesis (13.6%, three of 22 cases) (Fig. 2A) failed compared to only 2.8% (three of 106 cases) of the dedicated ramus plates (Fig. 2B) within the present study. However, compared to the BSSO group, where no such complication occurred (*p* = 0.042), the results of this study suggest that the risk of material failure is significantly higher for the HOO procedure, whereby the use of ramus plates seems to significantly reduce this risk and, thus, should be the preferred osteosynthesis material for the HOO.

#### Sensory disturbances

**Fig. 4:**
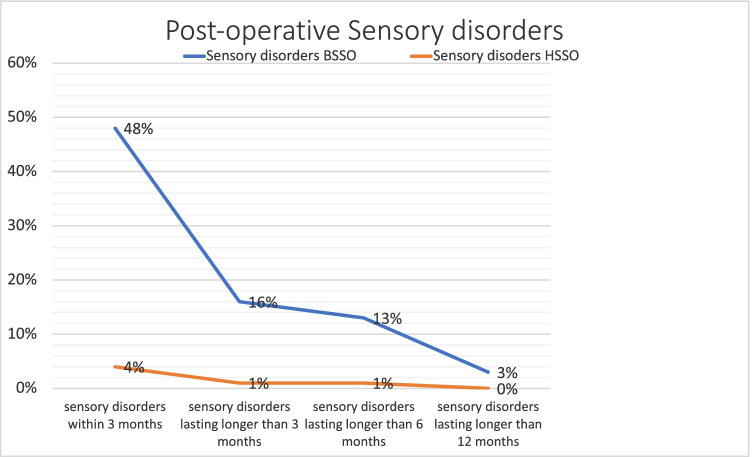
The percentage of patients presenting postoperative sensory disorders in forms of a hypaesthesia or paraesthesia directly, 3, 6, and 12 months after the operation

While the osteotomy line runs consistently above the mandibular foramen in the HOO procedure, the BSSO procedure poses an increased risk of damage to the inferior alveolar nerve due to the course of the osteotomy lines in the ascending mandibular branch and the posterior body of the mandible and in the course of the sagittal split. Direct or indirect mechanical traumatic events can lead to damage to the sensitive nerve fibres during or after the operation. [37] The neurosensory deficit is usually perceived by the patient as paraesthesia or as reduced sensitivity in the anatomical area innervated by the inferior alveolar nerve. [37] The prevalence of sensory disturbances is reported to be between 11.2 and 55% for the BSSO. [37,38] Hanzelka et al. showed that 48.3% of patients had sensory disturbances 4 weeks after surgery; however, this number declined to 3.1% 12 months after surgery, which is also reflected by the finding of our study, where 48.4% of the patients (44/91) in the BSSO group developed postoperative sensitivity disorders in forms of a hypaesthesia or paraesthesia, which declined to only 3.3% (3/91) after 12 months (Fig. 4). Compared to low number (3.9% (5/128)) of patients with sensory disturbances in the HOO group (*p* = 0.00), it can be assumed that the risk of developing sensory disturbances is significantly higher in the early postoperative phase when choosing the BSSO; however, no significant differences can be found for permanent sensory disturbances.

#### Infections, secondary bleeding, and other complications

Two of 291 patients (0.91%) (BSSO 1/91, 1.1%; HOO 1/128, 0.8%; *p* = 1) developed a postoperative infection, which required the removal of the osteosynthesis material in both cases. The reported incidences for infections after osteotomies of the mandible range between 0.7 and 10.2%. [39] With only two patients (BSSO 1/91, 1.1%; HOO 1/128, 0.8%; *p* = 1), our results can be located at the lower end of reported incidences. In this context, it must be highlighted that perioperative antibiotic prophylaxis can significantly reduce the risk of postoperative infections [40], which might have led to the low number of postoperative infections.

Postoperative bleeding is also described as a possible complication after orthognathic surgery, whereby these are generally associated more with the Le Fort 1 osteotomy. [36] In this study, one case from the BSSO group (0.78%) had recurrent bleeding after hospital discharge, which necessitated an emergency re-presentation and surgical intervention. In the HOO group, no case of secondary bleeding is documented (*p* = 0.41).

Moreover, unexpected perioperative complications can occur in the course of every operation, regardless of the respective surgical area or surgical procedure. In this study, a case of postoperative lactic acidosis in a type I diabetes patient (HOO group) and a compartment syndrome on the lower leg (HOO group) were documented as rare complications. In their study on rare complications following orthognathic surgery, Steel and Cope also report about the occasional development of a compartment syndrome. [41] Incorrect patient positioning and postoperative trauma are discussed as possible reasons. It should be noted, however, that such complications can always be related to the operation in a purely coincidental manner.

### Limitations

There are limitations to this retrospective study which have to be taken into consideration before interpretation. Patients with a syndrome, a systemic disease, and a TMJ disorder and patients with operative interventions in terms of a temporomandibular joint replacement have been excluded. Moreover, the extent of the transposition was not taken into account due to insufficient clinical data. As a result, the impact of this factor in terms of the postoperative stability was not included in our study. Furthermore, the surgeries have been carried out by different albeit experienced surgeons, which might limit the conclusions drawn to the operation method.

However, the analysis of an extensive patient collective can deliver an important and good overview regarding the advantages of both surgical techniques. Further studies with a prospective study design need to be conducted to investigate and compare the BSSO and HOO in more depth.

## Conclusion

In conclusion, our results show that there are significant differences regarding intra- and postoperative complication rates between the BSSO and HOO. The risk of developing intraoperative complications like a bad split seems to be higher for the BSSO due to the more complex osteotomy line of the mandible. Also, the risk of developing early postoperative sensory disturbances is significantly higher in the BSSO due to direct and indirect traumatic events that lead to damage to the sensitive nerve fibres; however, in the majority of patients, the sensory function recuperates in the long term. On the other hand, the risk of osteosynthesis material failure is significantly higher for the HOO due to the smaller bone apposition area and, thus, less stability of the re-fixation. Newly developed osteosynthesis materials, however, significantly reduce this risk. The BSSO still remains the surgical technique of choice especially for large anterior–posterior movements of the mandible due to the greater bone apposition area. For smaller anterior–posterior transpositions and (counter-)clockwise rotations, the HOO can provide a viable alternative to the BSSO.
